# Effect of Graphene Oxide on the Mechanical Property and Microstructure of Clay-Cement Slurry

**DOI:** 10.3390/ma16124294

**Published:** 2023-06-09

**Authors:** Shujie Liu, Jinze Sun, Jiwei Zhang, Zuodong Xie, Zhijie Yu

**Affiliations:** 1 China Coal Research Institute, Beijing 100013, China; 13681342073@163.com (S.L.); 15618536658@163.com (J.S.);; 2 Beijing China Coal Mine Engineering Company Ltd., Beijing 100013, China; 3 School of Civil and Resource Engineering, University of Science and Technology Beijing, Beijing 100085, China; 4 National Engineering Research Center of Deep Shaft Construction, Beijing 100013, China

**Keywords:** clay-cement slurry, graphene oxide, cement, micro structure

## Abstract

As a widely used material in underground engineering, clay-cement slurry grouting is characterized by poor initial anti-seepage and filtration capacity, low strength of the resulting stone body, and a tendency to brittle failure. In this study, a novel type of clay-cement slurry was developed by adding of graphene oxide (GO) as a modifier to ordinary clay-cement slurry. The rheological properties of the improved slurry were studied through laboratory tests, and the effects of varying amounts of GO on the slurry’s viscosity, stability, plastic strength, and stone body mechanical properties were analyzed. The results indicated that the viscosity of clay-cement slurry increases by a maximum of 163% with 0.05% GO, resulting in a decrease in the slurry’s fluidity. The stability and plastic strength of GO-modified clay-cement slurry were significantly enhanced, with the plastic strength increasing by a 5.62 time with 0.03% GO and a 7.11 time with 0.05% GO at the same curing time. The stone body of the slurry exhibited increased uniaxial compressive strength and shear strength, with maximum increases of 23.94% and 25.27% with 0.05% GO, respectively, indicating a significant optimization effect on the slurry’s durability. The micro-mechanism for the effect of GO on the properties of slurry was investigated using scanning electron microscopy (SEM) and a diffraction of X-rays (XRD) test. Moreover, a growth model of the stone body of GO-modified clay-cement slurry was proposed. The results showed that after the GO-modified clay-cement slurry was solidified, a clay-cement agglomerate space skeleton with GO monolayer as the core was formed inside the stone body, and with an increase in GO content from 0.03% to 0.05%, the number of clay particles increased. The clay particles filled the skeleton to form a slurry system architecture, which is the primary reason for the superior performance of GO-modified clay-cement slurry when compared with traditional clay-cement slurry.

## 1. Introduction

The hydraulic sealing and rock reinforcement effects of the grouting method are significant, and it is widely used in the field of underground engineering disaster management [1,2,3,4]. The selection of grouting materials as an important component of grouting reinforcement technology has a significant influence on the reinforcement effect [5,6,7]. With the development of grouting technology, various types of grouting materials have emerged, which are mainly divided into inorganic grouting materials and organic grouting materials. As an inorganic grouting material, bentonite cement slurry has been widely applied due to its excellent performance, which is characterized by the following attributes: (1) good fluid stability, with no segregation or precipitation during pumping and diffusion processes, less water separation and a high solidification rate during the consolidation process, and good impermeability; (2) fine bentonite particles, good slurry fluidity, and easy penetration into small fissures in rock formations; (3) strong plasticity and a large adjustable range of slurry strength, with adjustable and controllable slurry setting and hardening time, suitable for fissure grouting in different geological conditions; and (4) a mineral composition with good chemical inertness, strong resistance to underground water erosion, and good durability of the consolidated body [8]. However, bentonite cement slurry has a long setting time, low initial impermeability and filtration capacity, and low strength of the consolidated body, and it is prone to brittle failure, which affects the grouting reinforcement effect [9]. 

Graphene oxide, a derivative of graphene, has a large specific surface area, dispersibility, and excellent mechanical properties. Its layered surface has rich functional groups and, therefore, it has been applied to the modification of materials such as oxides and polymer polymers [10]. In recent years, many scholars have studied the reinforcing effect of graphene oxide on cement-based grouting materials.

Gao et al. [11] added carbon nanotubes and graphene oxide composite nanomaterials to cement-based slurries, and found that with the addition of composite nanomaterials, the impermeability, mechanical properties, and toughness of the cementitious body were greatly enhanced. Lu et al. [12] added 0.01%, 0.03%, and 0.05% graphene separately to ground cement materials and tested the compressive strength of the cement-based composite material at 3 d and 28 d, finding that the addition of graphene enhanced the compressive strength of the cement-based grouting material, and that adding 0.03% graphene resulted in the most obvious improvement on the strength of cement-based materials. Pan [13] added graphene oxide to 75 cement-based materials and found that 0.05 wt.% of graphene oxide increased the com-76 pressive strength of cement-based composite materials by 15% to 33%, and the flexural 77 strength by 41% to 59%. Yang et al. [14] found that adding 0.03% graphene oxide to cement mortar could achieve the highest corrosion resistance.

Mohammed et al. [15] found that adding graphene oxide to cement mortar could effectively prevent chloride ions from entering the sample and could effectively reduce water adsorption. Therefore, adding graphene oxide to cement mortar could improve its transport properties and increase the durability of the slurry. Horszczaruk [16] added graphene oxide to cement mortar and characterized the mortar’s micro-mechanical properties using AFM. The results showed that the Young’s modulus of the graphene oxide-containing mortar samples was distributed in the range of 5–20 GPa, while the control samples were distributed in the range of 1–10 GPa. Li et al. [17,18] prepared saturated Ca(OH)_2_ to simulate the alkaline environment in the hydration process of cement and mixed graphene oxide (GO) with Ca(OH)_2_ solution for stirring. By observing agglomerates through SEM, they found that the surface had wrinkles and folds, and the agglomerate size was much larger than that of the original graphene oxide flakes.

In order to improve the initial impermeability and strength of the clay-cement slurry, graphene oxide (GO) prepared by the Hummers method was used as a modifier, and a new type of clay-cement slurry was prepared through optimization methods such as vibration. Through indoor tests, the effect of GO addition on the main performance parameters such as viscosity, stability, plastic strength, and mechanical properties of the stone body of the clay-cement slurry was studied, and a growth model of the GO-modified clay-cement slurry stone body was proposed. The mechanism of GO action was analyzed from the perspective of micro-morphology. This paper aimed to provide a high-strength, high impermeability, and high durability grouting material for weakly cemented gravel layers and to provide scientific guidance for the proportioning of grouting materials.

## 2. Materials and Methods

### 2.1. Materials

#### 2.1.1. Clay-Cement Slurry

Clay-cement slurry is a multiphase suspension that is mainly composed of clay slurry, with a small amount of cement and water glass added. The plasticity index of the clay used should not be less than 10, the content of clay particles with a particle size smaller than 0.005 mm should not be less than 25%, and the sand content should not exceed 5%. The cement used should have a strength grade of not lower than 42.5, its fineness should meet the requirements of GB/T 1345 [19], and the residue on an 80 μm square-hole sieve should not exceed 5% [20].

In this experiment, pure calcium chloride was used as the main component, which is an inorganic powder that is usually stored in a sealed container due to its anhydrous nature. With small amounts of additives added, it can be configured into a single-liquid cement slurry that has good impermeability of the stone body, is non-toxic and harmless, has a simple process, and is easy to operate. However, it is easy to lose during the grouting process, prone to precipitation of water, and difficult to inject into cracks smaller than 0.1 mm. Its main components are shown in Table 1.

**Figure 1:**
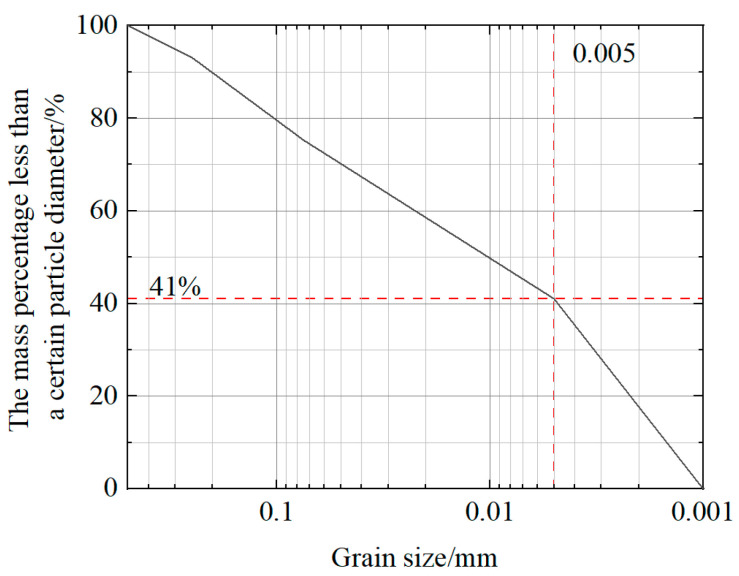
Clay particle size distribution curve.

In this study, quaternary clay from Henan Province, China, was mixed with water and left to soak for 2–3 days until fully infiltrated, then made into a slurry using a high-speed mixer, sieved through a 0.05 mm sieve to remove sand, and dried to obtain the clay stone body [20]. After crushing, it was sieved through a 0.05 mm sieve and the particle size composition was 6.9% for the 0.5–0.25 mm particle size, 17.8% for the 0.25–0.075 mm particle size, 34.3% for the 0.075–0.005 mm particle size, and 41.0% for the <0.005 mm particle size, as shown in the Figure 1, which met the requirements. The analysis results of clay properties are shown in Table 2.

#### 2.1.2. Graphene Oxide

GO suspension (2 mg/mL) is an industrial-grade, single-layer graphene oxide dispersion prepared by Suzhou Carbon Century Graphene Technology Co., Ltd. (Suzhou, China), of China using an improved Hummer’s method. The diameter distribution of 3~8 nm accounted for 95% of the suspension, a thickness less than 3 nm accounted for 90%, and the purity reached 99%.

### 2.2. Methods

#### 2.2.1. Sample Preparation

The density of the clay slurry was set at 1.15 g/cm^3^, with a total mixing water-to-cement ratio of 5%, 10%, and 15% of the clay slurry volume, and a mixing water-to-sodium silicate ratio of 10 mL/L. In order to induce wrinkles and folds in the graphene oxide (GO), the GO dispersion was mixed with ordinary Portland cement (OPC) and stirred thoroughly before adding it to the clay slurry mixture. Sodium silicate was then added to promote cement hydration and save time, and the resulting slurry was poured into a mixer for further stirring. The amount of GO dispersion used was 0%, 0.01%, 0.02%, 0.03%, 0.04%, and 0.05% of the cement mass. 

**Figure 2:**
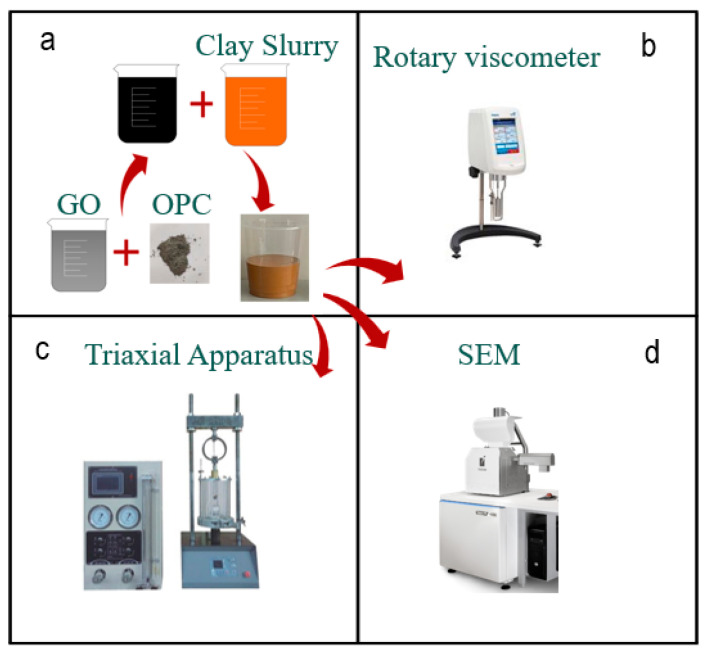
Test method and process: (**a**) preparation of graphene oxide modified clay cement slurry; (**b**) viscosity test; (**c**) strength test; (**d**) electron microscopy scanning.

The clay was dried in a 105 °C drying oven for one day, cooled to room temperature, then ball-milled for 30 min to form a powder before being weighed. The clay was then mixed with a solution of GO and stirred for 5 min. The cement and mineral admixture were weighed and uniformly mixed before being poured into the clay—graphene suspension. The mixture was stirred for 5 min to form a homogeneous slurry. The detailed preparation process is shown in Figure 2. After preparation, the slurry was poured into a 70 × 70 × 70 mm mold, flattened, and placed in a curing box. The curing box had a temperature of 20.5 °C and relative humidity of 95%. Performance tests were conducted after 7 days of curing [20].

#### 2.2.2. Testing Procedures

The viscosity test was conducted using a WT-AUTO-900 rotary viscometer. To avoid the influence of standing time, the test was started 10 min after the slurry was prepared. The water separation rate of the slurry was determined using a 100 mL graduated cylinder. One hundred mL of the slurry was poured into the cylinder, covered with plastic wrap, and left to stand for 30 min. The water separation rate was then calculated to evaluate the stability of the slurry, with a slurry water separation rate of 5% used as the stability indicator. After standard curing in a standard curing box, the plastic strength of the material was measured using an improved Vicat apparatus, following the water consumption, setting time, and stability test methods in GB/T 1346-2011 [21]. The compressive strength and shear strength of the stone bodies at different ages were tested according to the “Cement Mortar Strength Test Standard Cement Mortar Strength Test Method (ISO Method)” (GB/T 17671-1999) [22]. The microstructure of the material was analyzed using a TESCAN VEGA scanning electron microscope.

## 3. Results

### 3.1. Slurry Injectability

#### 3.1.1. Slurry Rheology

**Figure 3:**
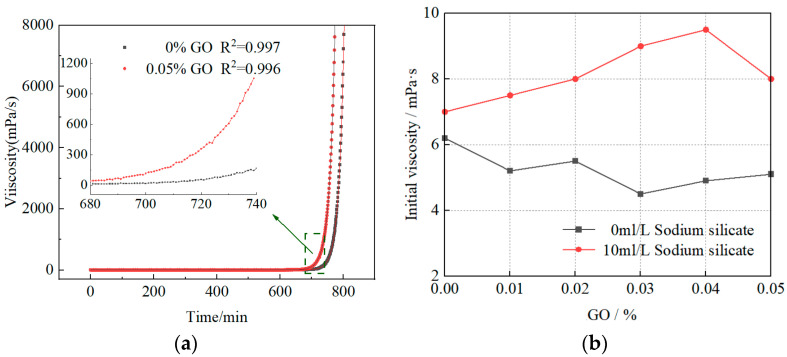
Effect of GO dosage on slurry viscosity: (**a**) viscosity time-varying (100 g/L OPC); (**b**) initial viscosity (100 g/L OPC).

The slurry viscosity reflects the fluidity of the slurry and can provide reliable reference for slurry selection at the grouting site. The effect of GO content on slurry viscosity is shown in Figure 3.

The time-dependent curve of the slurry shown in Figure 3a can be divided into three parts. First, during the initial testing phase, the slurry viscosity remains stable due to an incomplete cement-hydration reaction, an intact clay-slurry structure, and low inter-particle friction. In the second stage, the slurry viscosity shows an exponential increase. During cement hydration, a portion of the free water is absorbed and a large number of clay particles are electrostatically adsorbed on the surface of cement hydration products, forming large volume agglomerates, which results in a significant decrease in slurry fluidity. In the third stage, the cement hydration process is essentially completed, the viscosity rapidly increases, and the slurry loses its fluidity. This is mainly due to water molecules trapped in the aggregates of nano materials, which is caused by the van der Waals force and electrostatic interaction between GO flakes, which then reduces the amount of free water available [23]. Moreover, the extremely high specific surface area of GO flakes adsorbs a large amount of free water [24], resulting in a reduction in particle spacing and the enhancement of friction between particles in the system, which is manifested as an increase in shear stress in a macro-scopic view.

In addition, the inflection point of the viscosity curve of the clay-cement slurry doped with oxidized graphene compared with the control group is ahead, which indicates that GO can promote the cement hydration reaction, accelerate the volume and rate of agglomeration of clay-cement slurry particles, and inhibit the fluidity of clay-cement slurry in the early stage. Furthermore, as shown in Figure 3b, GO has no significant influence on the initial viscosity of the clay-cement slurry.

Further analysis of Figure 3a reveals that the increase in viscosity during the agglomeration process of the oxidized graphene clay-cement slurry and the traditional clay-cement slurry over time follows the pattern shown in Figure 3a. The fitting curve exhibits an exponential distribution and the fitting functions can be expressed, respectively, as:

(1) $$\mu =1.66\cdot 10^{\left(-16\right)}\cdot \exp\left(\frac{t+3.44}{17.15}\right)+7.84,R^{2}=0.996$$

(2) $$\mu =2.03\cdot 10^{\left(-18\right)}\cdot \exp\left(\frac{t}{16.15}\right)+5.93,R^{2}=0.997$$

In the formula: *μ* represents the viscosity (mPa · s) and t represents the mixing time (min).

There are two reasons why the addition of GO reduces the fluidity of the slurry. First, GO provides growth sites for hydration products, forming larger cement agglomerates. Smaller clay particles are adsorbed around cement particles by electrostatic effects, resulting in larger clay-cement particle agglomerates and increasing the mechanical friction effect between clay particles, leading to a decrease in slurry fluidity. Second, GO has hydrophilic properties, and the amount of water consumed gradually increases with the increase in GO content, which reduces the content of water molecules bound to cement and consequently reduces the fluidity of the slurry.

#### 3.1.2. Slurry Bleeding Rate

The slurry bleeding rate is an important indicator for evaluating the stability and pumpability of the slurry. It also affects the structure of the stone and the reinforcement effect of grouting. The lower the slurry bleeding rate of the slurry, the less prone it is to segregation and stratification and the more suitable it is for pipeline transportation. It has a better effect on filling cracks or pores and leads to a lower rate of water seepage in the later stages of the stone formation.

**Figure 4:**
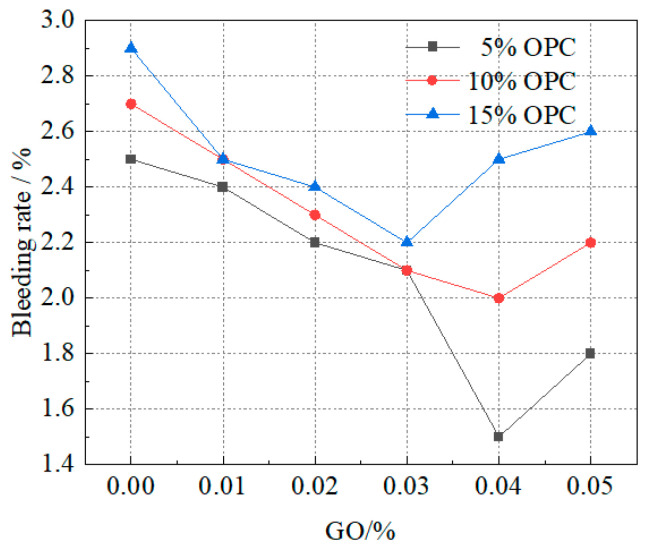
Effect of GO and OPC on water separating rate.

The influence of each component on the slurry bleeding rate is shown in Figure 4. It can be seen that GO had an inhibitory effect on the water loss rate of the slurry. When 100 g/L of OPC was added without water glass, the slurry bleeding rate gradually decreased with the increase in GO content from 0.01% to 0.03%, then slowly increased before stabilizing. When 10 mL/L of water glass was added, the slurry bleeding rate increased with the increase in OPC content. The results indicated that a small amount of GO has an inhibitory effect on the slurry bleeding rate, which gradually weakens with the increase in GO content. 

Wang et al. [25] added 0.05% graphene oxide to cement slurry and studied its flow properties, finding that the flowability of cement slurry decreased, viscosity increased, and cement setting time shortened. It is noteworthy that when the amount of graphene oxide was 0.03%, the changes in flow properties, viscosity, and setting time of cement slurry were significant, which could promote the hydration of cement slurry.

When the OPC content was 5% and 10% and the GO content was 0.04%, the slurry bleeding rate was the lowest at 1.1% and 2.0% respectively. When the clay content was 15% and the GO content is 0.03 wt.%, the bleeding rate was the lowest at 2.2%. A small amount of GO had an inhibitory effect on the slurry bleeding rate, which gradually weakened with the increase in GO content. The specific reasons were that (1) the hydrophilicity of GO reduced the water loss rate of the slurry to a certain extent and (2) GO was used as a growth site, forming larger clay-cement particle agglomerates that settled under gravity, thereby increasing the water loss rate of the slurry.

### 3.2. Slurry Strength

#### 3.2.1. Plastic Strength of Slurry

The strength of the slurry was measured using a Vicker cone penetrometer, in which the clay slurry was placed in a 65 × 75 × 40 mm sized conical mold. The mold was kept in the SBY-32B constant temperature cement curing box and the plastic strength of different slurry ratios was measured at regular intervals. The specific formula was as follows:

(3) $$P=\cos^{2}\left(\frac{\alpha }{2}\right)G/\pi \sin\left(\frac{\alpha }{2}\right)h^{2}$$

where *G* is the weight of the entire cone, α is the cone angle, and *h* is the distance that the cone penetrates the slurry. 

**Figure 5:**
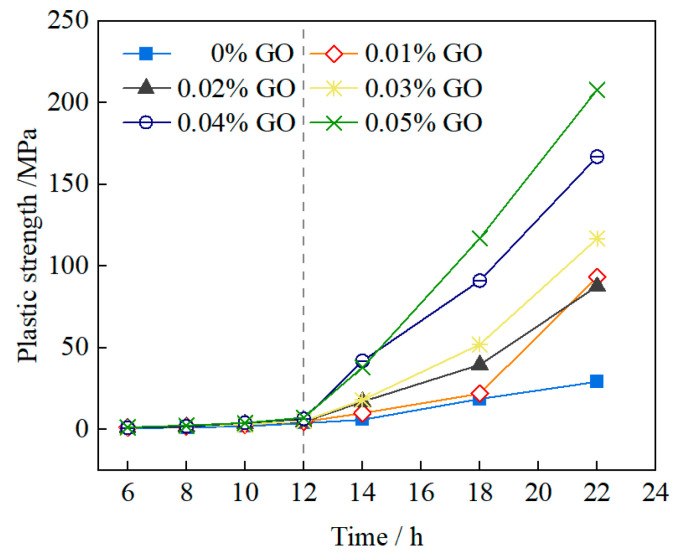
Effect of GO dosage on time-dependence of plastic strength (OPC of 150 g/L, water glass of 10 mL/L).

The plasticity feature is the late effect of the rheological characteristics of the slurry, and it plays a crucial role in resisting infiltration and water plugging. The results of the slurry’s plastic strength are shown in Figure 5. The following can be observed: (1) the plastic strength of the slurry with different mix ratios exhibited the same trend. Before 12 h, the plastic strength remained at a relatively low level, gradually increasing moderately, and then explosively increasing exponentially after 14 h; (2) compared with the control group, the plastic strength of the slurry significantly increased with the increase in GO content from 0.01% to 0.05% after 12 h, indicating that GO plays an optimal role in the early-stage water plugging performance of the slurry, consistent with the rules of the viscosity thixotropy mentioned earlier.

#### 3.2.2. Yield Strength of Slurry

**Figure 6:**
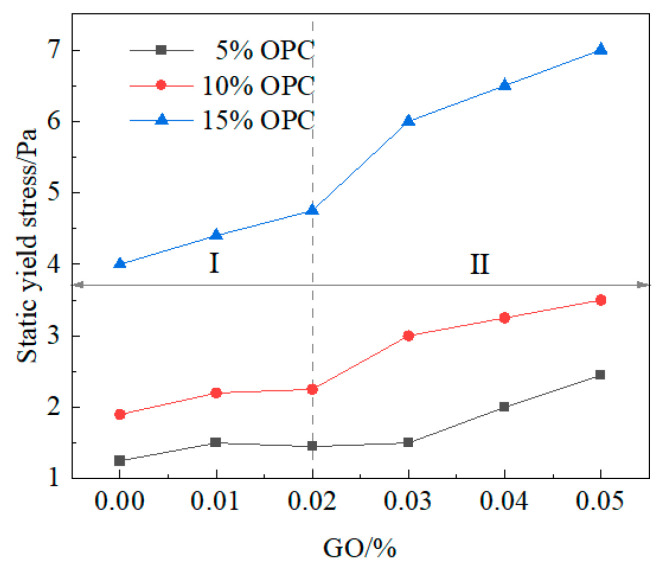
Yield stress of the slurry after being statically placed for 90 min.

For waterproof and anti-seepage engineering, a higher increase rate in the yield stress of the slurry material leads to stronger resistance against water erosion. In this section, we study the distribution of the yield stress values of different proportioned clay-cement slurries after being statically placed for 90 min. As shown in Figure 6, the yield stress of the three types of OPC content slurries under different GO ratios exhibited the same changing pattern, which suggests that the yield stress increased with the increase in GO content from 0.01% to 0.05%. Specifically, the yield stress increased significantly with GO content above 0.02%, whereas it only showed a slight increase in the range of 10% to 18.75% when GO content was below 0.02%. The specific reason may be that GO accelerated the hydration process of cement, increased the volume of flocculated particles, and enhanced the overall performance of the slurry in terms of yield stress.

#### 3.2.3. Uniaxial Compressive Strength and Shear Strength of Slurry Rock Bodies

**Figure 7:**
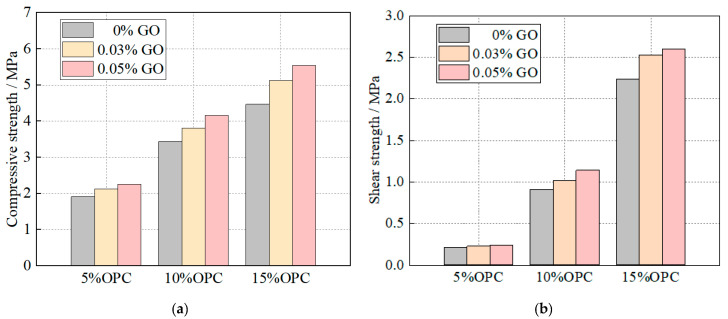
Effect of GO and OPC content on strength of the specimens (water glass of 10 mL/L): (**a**) compressive strength; (**b**) shear strength.

Figure 7a shows the uniaxial compressive strength of specimen 7 d. It can be observed that when the OPC content was constant, the compressive strength of the specimen increased with the increase in GO content. Similarly, when the GO content was constant, the compressive strength of the specimen increased with the increase in OPC content. The increase in compressive strength of the compact body with the increase in OPC content became more significant when the GO content was kept constant. For example, in the slurry content was 0.05% GO, the increase in compressive strength with the increase in OPC content was from 17.80% to 20.93% and 23.94%, respectively. The maximum increase in compressive strength was observed at 23.94%, when the OPC content was 15% and the GO content was 0.05%.

Figure 7b shows the effect of GO and OPC content on the shear strength of the specimen. It can be seen that the addition of GO significantly improved the shear strength of the specimen and the effect became more significant with the increase in OPC content. The addition of GO could transfer more cement hydration products into microcapillary pores and bridge the microcracks in the material, thus improving the shear strength of the material. This is because GO could transfer more cement-hydration products into the microcapillary pores of the material and bridge the microcracks, thereby improving the shear strength of the material [26]. The addition of 0.03 wt.% and 0.05 wt.% of GO resulted in an increase in the shear strength of the specimen by 9.52%, 12.08%, 12.95%, 14.29%, 25.27%, and 16.07%, respectively. This phenomenon indicated that GO increased uniaxial compressive strength and shear strength, as had already been reported for other cement-based materials [25,27,28].

### 3.3. Microstructure of Modified Clay-Cement Slurry Compact Body

#### 3.3.1. Microstructure Analysis of Modified Clay-Cement Slurry Compact Body

**Figure 8:**
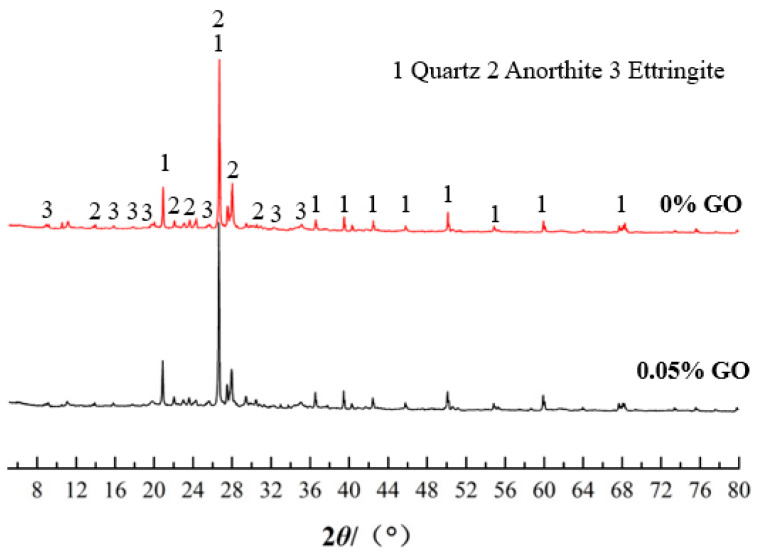
X-ray diffraction pattern.

The content of GO with 0% and 0.05% were tested via XRD between 5° and 80° after 7 d of curing. The X-ray diffraction spectra are shown in Figure 8. Diffraction peaks of quartz and ettringite were observed in the figure. The diffraction peak of calcian feldspar also appeared in the spectra, which is a hydrated product of the sample chemical reaction. The formation of calcian feldspar is crucial to enhance early strength. It can be seen that there was no significant difference in the diffraction peaks of the two samples’ XRD spectra, whether or not GO was added. The research of Balaji and Yang [29,30] showed a similar situation. This is mainly due to the fact that GO accelerates the hydration process but does not change the types of hydration products. The recognition peak of GO is not visible in the XRD diffraction pattern because of the very small amount of GO and the very high strength of the crystalline phase composition of the clay [31,32].

**Figure 9:**
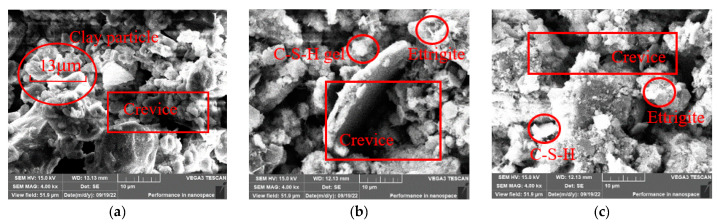
Microstructure of specimens with GO content of 0: (**a**) OPC 5%; (**b**) OPC 10%; (**c**) OPC 15%.

**Figure 10:**
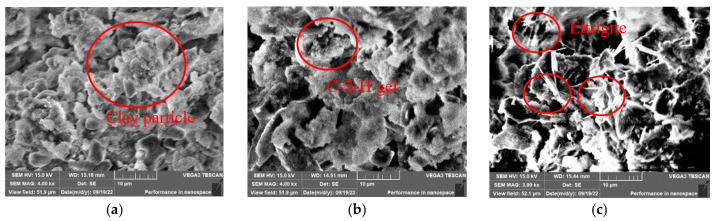
Microstructure of specimens with GO content of 0.03%: (**a**) OPC 5%; (**b**) OPC 10%; (**c**) OPC 15%.

**Figure 11:**
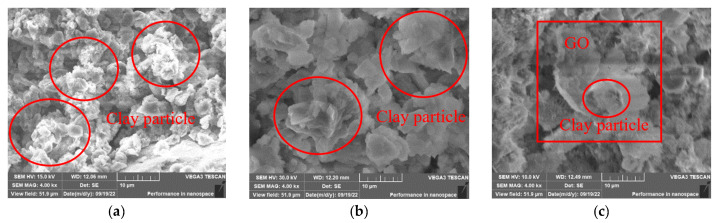
Microstructure of specimens with GO content of 0.05%: (**a**) OPC 5%; (**b**) OPC 10%; (**c**) OPC 15%.

To observe the microstructure and morphology of the modified clay-cement slurry compact body with three different percentages of GO content after seven days of curing, SEM analysis was performed. The results are shown in Figure 9, Figure 10 and Figure 11.

Figure 8 shows the microstructure of the slurry compact body with different OPC content when the GO content is 0%. When GO is not doped, some hydration products, mainly rod-shaped and needle-shaped ettringite that react rapidly, are produced. In addition, there are small amounts of C-S-H gel and calcium hydroxide crystals. When the slurry contains 5% OPC, with an increase in cement content, a large amount of ettringite and C-S-H gel hydration products generated by cement hydration become a skeleton, which further bonds under electrostatic attraction with clay-cement agglomerates and begins to fill in inter-particulate voids. The agglomerates have a discontinuous structure with numerous pores and are relatively loose. Some of the hydration products are not enveloped by clay particles and are exposed to the exterior.

Figure 10 shows the microstructure of the slurry compact body with different OPC content when the GO content is 0.03%. Comparing Figure 10a with Figure 9a, it can be observed that the cement hydration products are completely wrapped around the clay particles, and the volume of the clay-cement aggregate increases. The internal voids in the system are filled and bonded, forming a dense structure. When the OPC content is 10% and 15%, the cement hydration products are completely wrapped around the clay particles without any obvious exposure. However, when the OPC content is 15%, some hydration products are not completely wrapped around, and the addition of GO can transfer more cement hydration products into microcapillary pores and bridge the microcracks in the material, thus improving the material properties. Such a structure provided greater help in the improvement of the physical and mechanical properties of the sample, a result that is similar to the results of other studies [33].

Figure 11 shows the microstructure of the slurry compact body with different OPC content when the GO content is 0.05%. Compared with Figure 10a, it can be observed that, with the increase in GO content, there is no obvious change in the volume of the clay-cement aggregate, but the number of aggregates increases. The skeleton strength of the aggregate-clay particle filling system improves, which is manifested as an increase in the compressive and shear strength of the material, consistent with the experimental results. Comparing Figure 9b, Figure 10b and Figure 11b, it can be observed that as the GO content increases, the cement hydration products grow around its oxygen-containing functional group and are wrapped around the clay, resulting in a decrease in the exposed surface area and the gradual densification of the surface structure. Figure 11c shows the plate-like graphene oxide that is not completely wrapped around the clay particles and the clay-cement particles that grow around its oxygen-containing functional group and are wrapped by the clay particles. This indicates that the aggregate-clay particle filling system is not completely formed at this time, resulting in a smaller increase in the shear and compressive strength of the compact body with 0.05% GO content, as compared to that with 0.03% GO content.

Based on the above results, it can be concluded that with the increase in GO content, the volume of the aggregates increases, the number of cracks decreases significantly, and the structural density increases. This indicates that the addition of GO can optimize the pore size distribution and improve the performance of the material.

#### 3.3.2. Microscopic Growth Process Model of Slurry Concretion

**Figure 12:**
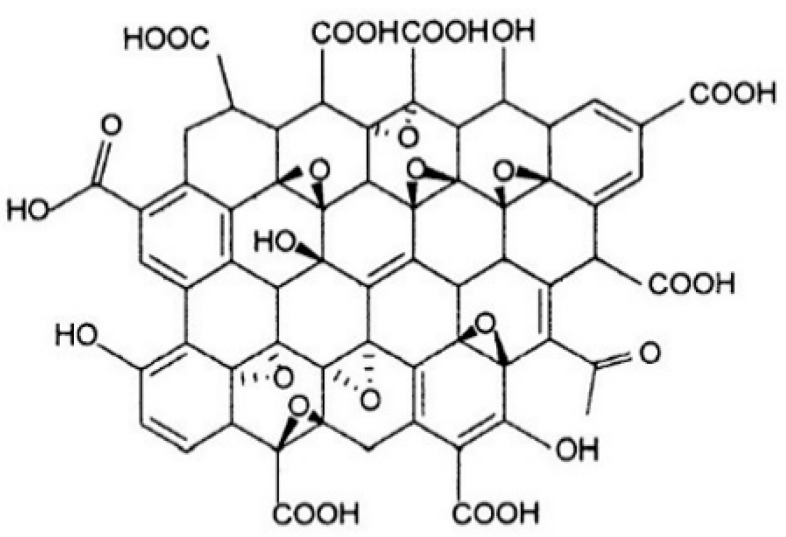
GO structure diagram.

Traditional clay-cement slurry produces cement particles with a positive charge during cement hydration. The cement particles undergo partial charge exchange with clay particles, and because cement particles are larger than clay particles, clay particles are adsorbed onto the surface of cement particles and agglomerated, forming a gelatinous skeletal structure with some strength in an alkaline environment. Research indicates that there are two main mechanisms for reinforcing the micromechanical structure of cementitious composites and enhancing their performance with nanomaterials such as carbon nanotubes, graphene, and graphene oxide [15]. After graphene oxide is added to clay-cement slurry, on the one hand, the nanomaterial itself has a nucleation effect, promoting cement hydration reaction and further forming C-S-H with more cement hydration products that can transmit in the capillary pores. On the other hand, the nanomaterial acts as a bridge in the microcracks of the material [34]. The molecular structure of the GO is shown schematically in Figure 12 [35].

**Figure 13:**
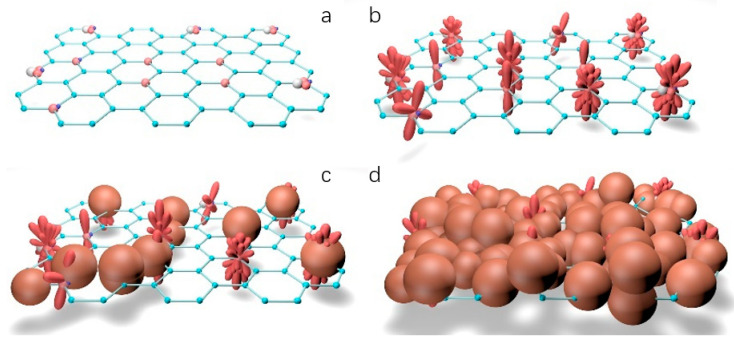
Microscopic growth process model of stone body: (**a**) graphene oxide structure; (**b**) cement hydration process; (**c**) clay adsorption process; (**d**) clay-cement particle agglomerate skeletal structure.

Based on the SEM scan results in the previous section, as the GO content increases, the volume of the clay-cement particle agglomerate increases. Meanwhile, as shown in Figure 11c, cement agglomerates grow on the layered graphene oxide without being completely wrapped by clay particles. The following growth model is proposed: the layered GO structure serves as a growth point for cement such as C_3_S, C_2_S, and C_4_AF [36,37] and promotes the growth of subsequent hydration products, resulting in the formation of larger cement-particle agglomerates with a positive charge, which undergo partial charge exchange with negatively charged clay particles in the slurry. As cement particles are larger than clay particles, clay particles are adsorbed onto the surface of cement particles and agglomerated to form a clay-cement particle agglomerate with a GO layer as the core in an alkaline environment. Clay particles fill the voids in this agglomerate, forming a compact structural framework and increasing the density of the slurry system. This improves the compressive strength of the slurry stone body and is consistent with experimental results [38]. On the other hand, GO can transmit more hydration products into capillary pores and act as a bridge to connect microcracks in the material, thereby improving the shear resistance of the material. Figure 13 shows the process model of clay-cement slurry stone body growth modified by graphene oxide.

## 4. Conclusions

In this paper, different contents of GO were added to clay–cement grouting material. Several tests and characterization methods were used to investigate the influence of GO concentration on the viscosity, stability, plastic strength, and stone body mechanical properties and microstructure of GO grouting materials. The following conclusions were drawn:

(1) GO has an inhibitory effect on the early fluidity of clay-cement slurry but has no significant effect on the initial viscosity of the slurry. The viscosity of graphene oxide-clay-cement slurry follows an exponential distribution with time. A small amount of GO has an inhibitory effect on the slurry bleeding rate, which gradually weakens with increasing GO content;
(2) The plastic strength curve of the slurry with different mix ratios shows the same trend. Before 12 h, the plastic strength of the slurry increases slightly with the increase in GO content. After 14 h, the plastic strength of the clay-cement slurry increases significantly. This indicates that GO has an optimizing effect on the water plugging performance of the slurry in the early stage. Under different GO ratios, the static yield stress, uniaxial compressive strength, and shear strength show the same variation pattern, i.e., the yield stress increases with the increase in GO content, especially when the production rate is above 0.02%, when the increase in yield stress is significantly enhanced;
(3) The addition of GO promotes the generation of large-volume clay-cement particle agglomerates, resulting in a compact agglomerate skeleton structure filled with clay particles. This is the main reason for the improvement of material mechanical properties.

## Figures and Tables

**Table 1:** Chemical composition of test cement.

Chemical Composition	SiO_2_	Al_2_O_3_	Fe_2_O_3_	CaO	MgO	SO_2_
Value/%	21.32	4.31	3.38	61.26	2.47	2.55

**Table 2:** Basic parameters of clay.

Property	Water Content (%)	Plastic Limit (%)	Liquid Limit (%)	Plasticity Index	Liquid Index
Value	3.7	24.2	41.1	17.2	−1.19

## Data Availability

The data used to support the findings of this study are included within the article.
